# Evolution of Acyl-Substrate Recognition by a Family of Acyl-Homoserine Lactone Synthases

**DOI:** 10.1371/journal.pone.0112464

**Published:** 2014-11-17

**Authors:** Quin H. Christensen, Ryan M. Brecht, Dastagiri Dudekula, E. Peter Greenberg, Rajesh Nagarajan

**Affiliations:** 1 Department of Microbiology, University of Washington, Seattle, Washington, United States of America; 2 Department of Chemistry and Biochemistry, Boise State University, Boise, Idaho, United States of America; University of Freiburg, Germany

## Abstract

Members of the LuxI protein family catalyze synthesis of acyl-homoserine lactone (acyl-HSL) quorum sensing signals from *S*-adenosyl-L-methionine and an acyl thioester. Some LuxI family members prefer acyl-CoA, and others prefer acyl-acyl carrier protein (ACP) as the acyl-thioester substrate. We sought to understand the evolutionary history and mechanisms mediating this substrate preference. Our phylogenetic and motif analysis of the LuxI acyl-HSL synthase family indicates that the acyl-CoA-utilizing enzymes evolved from an acyl-ACP-utilizing ancestor. To further understand how acyl-ACPs and acyl-CoAs are recognized by acyl-HSL synthases we studied BmaI1, an octanoyl-ACP-dependent LuxI family member from *Burkholderia mallei*, and BjaI, an isovaleryl-CoA-dependent LuxI family member from *Bradyrhizobium japonicum*. We synthesized thioether analogs of their thioester acyl-substrates to probe recognition of the acyl-phosphopantetheine moiety common to both acyl-ACP and acyl-CoA substrates. The kinetics of catalysis and inhibition of these enzymes indicate that they recognize the acyl-phosphopantetheine moiety and they recognize non-preferred substrates with this moiety. We find that CoA substrate utilization arose through exaptation of acyl-phosphopantetheine recognition in this enzyme family.

## Introduction

Bacterial quorum sensing is a genetic regulatory phenomenon whereby cells excrete or secrete a chemical signal into the surrounding environment and at sufficient concentrations the signal alters expression of specific genes [1]–[3]. Many Proteobacteria use acyl-homoserine lactones (acyl-HSLs) as quorum sensing signals. Knowledge of acyl-HSL quorum sensing has been applied in many synthetic biology studies [4], and different strategies to evolve acyl-HSL synthases have been employed [5]–[7]. Because acyl-HSL quorum sensing affects the virulence of some bacterial pathogens, there have been many efforts to identify inhibitors of acyl-HSL receptor proteins, acyl-HSL synthases, or both [8]–[11].

**Figure 1:**
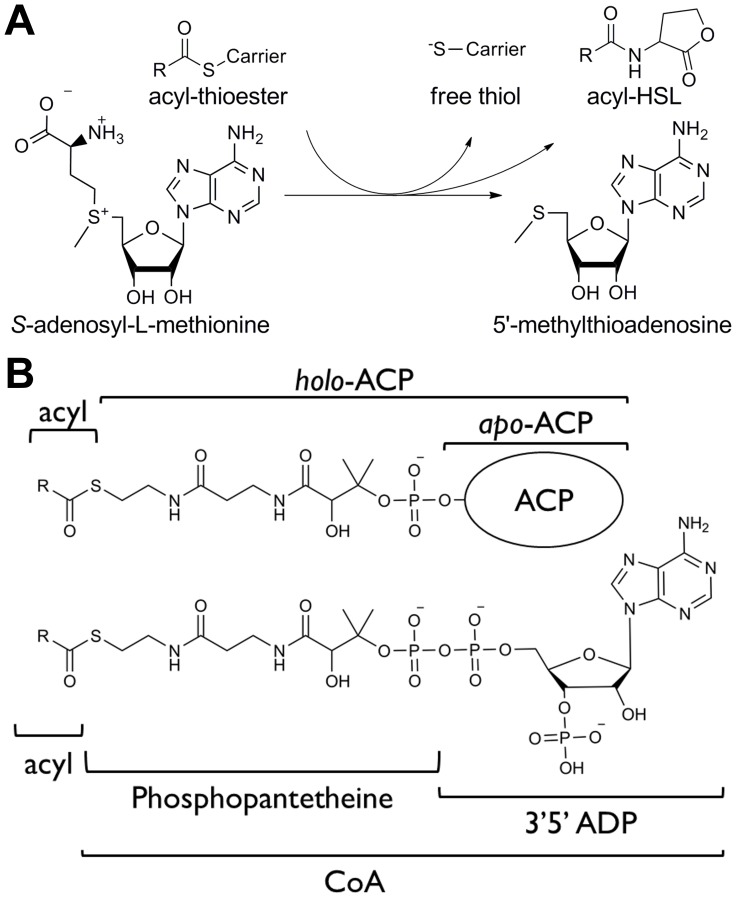
Substrates and products of acyl-HSL synthases. A) Acyl-HSL synthases have two substrates and three products. The substrate acyl group is attached as a thioester to an acyl carrier: either an acyl carrier protein or coenzyme A**.** B) Comparison of the structures of acyl-ACP and acyl-CoA. Both carriers have an acyl-phosphopantetheine (acyl-PPant) moiety. Thioether analogs of these thioester substrates lack the acyl oxygen.

Most known acyl-HSL synthases (EC 2.3.1.184) are members of the LuxI protein family (PF00765), although nonhomologous isozymes do exist [12]. The substrates for acyl-HSL synthases are *S*-adenosyl-L-methionine (SAM) and an acyl-thioester in the form of an acyl-acyl carrier protein (ACP) intermediate of fatty acid biosynthesis [13], [14], or as has been shown recently for some acyl-HSL synthases, acyl-Coenzyme A (acyl-CoA) [15]–[17] (Fig. 1). The crystal structures of three acyl-ACP-dependent acyl-HSL synthases have been solved [10], [18], [19], and it is apparent from the structures that these three enzymes are part of the Gcn5 N-acetyltransferase (GNAT) superfamily, all of which share a common phosphopantetheine (PPant) binding fold [18], [20]. Structural comparisons and mutagenesis studies indicate that acyl-ACP-utilizing acyl-HSL synthases interact with ACP using a conserved, positively charged, helix [18], [19]. Little is known about how acyl-homoserine lactone synthases interact with their acyl-substrates or how ACP and CoA-utilizing types are related to each other [15]–[17].

Evolution of new enzyme activities can occur through gene duplication and amplification [21]–[23]. It is accepted from studies of natural and engineered enzyme evolution that changes in the core catalytic function of an enzyme occur rarely, and changes in substrate use and the resulting products occur more frequently [22], [24]. In many models, the process of gene amplification allows an ancestrally non-preferred substrate to be used, thereby providing an opportunity for that activity to become the new primary activity for that lineage [21]. Such substrate switching events are more accurately described as exaptation instead of adaptation. Adaptations are features that enhance fitness and were produced by natural selection for their current role, whereas exaptations are not produced by natural selection for their current role [25], but rather co-opted to solve a new problem. An example of a molecular exaptation comes from evolution of light-refracting lens crystallin proteins used for vision that were exapted from enzymes [26]. Crystallins used to have an enzymatic function, but the entire protein was exapted for the optical properties of the crystalline aggregate. Considerable potential for exaptation has been found in catabolic pathways [27] as well as in the broad specificity of many enzymes [28].

In this paper we describe an evolutionary event where a new type of acyl-homoserine lactone synthase arose through changes in substrate recognition. We know that acyl-ACP and acyl-CoA substrates have an acyl-PPant moiety in common (Fig. 1). By using a functional phylogenomic approach [29] we performed phylogenetic, motif, and kinetic analyses of acyl-HSL synthases. Our work indicates known acyl-CoA-utilizing acyl-HSL synthases evolved from an ancestral acyl-ACP-utilizing enzyme through application of acyl-PPant recognition to acyl-CoA substrates. As acyl-PPant recognition was not originally selected for acyl-CoA substrates, we find this as an example of an evolutionary exaptation event [25].

## Results

### Phylogeny of the LuxI-family of acyl-HSL synthases

**Figure 2:**
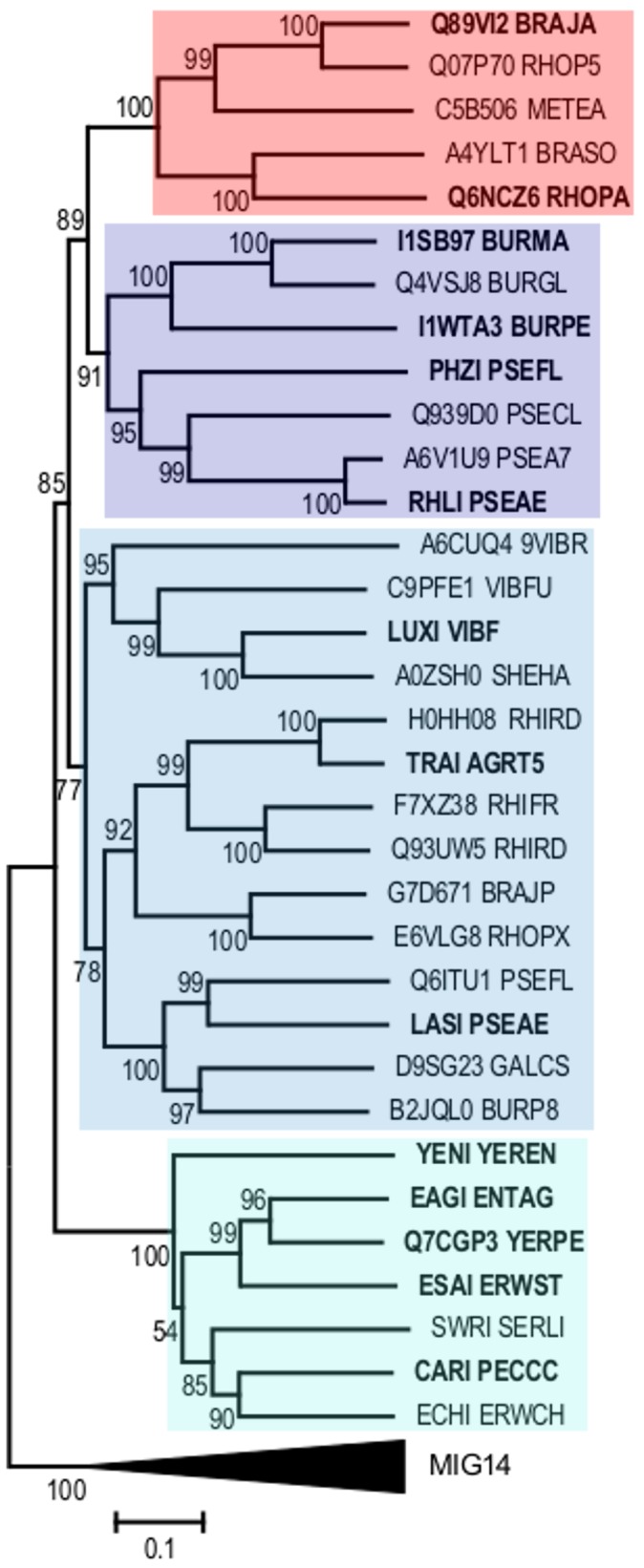
Protein phylogeny of acyl-HSL synthases from Pfam PF00765. The sequences used in the analysis are labeled with the uniprot identifier followed by the organism identifier. BmaI1 is I1SB97_BURMA and BjaI is Q89V12_BRAJA. The clade containing CoA-utilizing acyl-HSL synthases is highlighted in red and the clades containing acyl-ACP-utilizing acyl-homoserine lactone synthases are highlighted in shades of blue. The Mig14 family (PF07395), also from the acetyltransferase-like clan (CL0257), was used as an outgroup and is collapsed as a black triangle. Labels in bold have been experimentally shown to use ACP or CoA substrates. The percentage that each branch was observed during bootstrap resampling is shown next to the branch.

**Figure 3:**
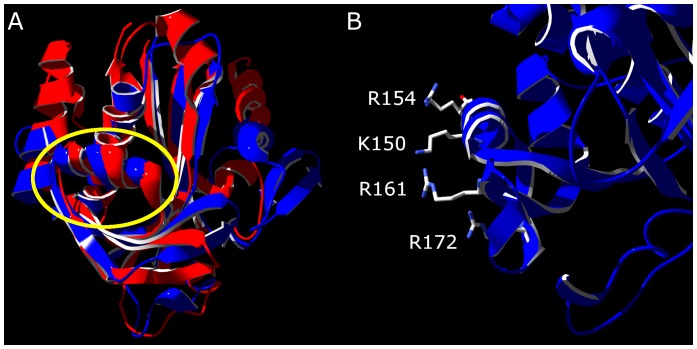
Structures of the acyl-substrate recognition motif. A) Alignment of the crystal structures of LasI (1R05 in blue) [18] and EsaI (1KZF in red) [19]. The two structures have a root-mean-square deviation of 1.45 Å for 124 amino acid α carbons. The conserved α-helix proposed to interact with ACP is circled in yellow. The active site cleft is behind this helix next to the conserved β-sheet. B) The LasI structure rotated 90° about the Z axis with positively-charged residues in the motif displayed.

To gain insight into the relationship between acyl-HSL synthase function and ancestry we constructed a phylogenetic tree by using the polypeptide sequences of diverse LuxI family members (Fig. 2). Previous LuxI family phylogenies were published prior to the discovery of acyl-CoA-dependent acyl-HSL synthases [30], [31]. Our tree is rooted close to the clade containing EsaI. In a previous phylogenetic analysis EsaI and relatives were put in a separate family [31]. We have since learned that the structures of EsaI and LasI are remarkably similar and there are conserved functional residues in the two proteins [18], [20], [19] (Fig. 3). These findings support our hypothesis that EsaI and LasI are homologs, and we include them together in our phylogenetic analysis. We rooted the tree to a member of the larger superfamily of GNAT acyltransferases (CL0257) as an outgroup [18], [20]. This allowed us to infer the evolutionary history of acyl-ACP recognition. The topology of the tree did not change with an alternate outgroup, with different types of type of distance matrices, or when using Maximum Likelihood or Minimum Evolution phylogenetic methods.

All known acyl-CoA-utilizing acyl-HSL synthases are grouped in a single clade and therefore can be described as monophyletic. This clustering indicates all known acyl-CoA-dependent synthases evolved from a common ancestor. In contrast, acyl-ACP-utilizing acyl-HSL synthases are found in every other clade in the family and so are paraphyletic. The most parsimonious interpretation of acyl-HSL synthase evolutionary history is one where the acyl-CoA-utilizing acyl-HSL synthase clade evolved once from an acyl-ACP-utilizing ancestor.

To illustrate our point we consider a couple alternate scenarios. First, if acyl-CoA-utilizing enzymes evolved in parallel with acyl-ACP-utilizing enzymes, we would expect the BjaI clade to be connected closer to the root of the tree than the other clades. We do not observe this with different methods of phylogenetic tree construction or with different outgroups determining the root. Second, if the common ancestor was an acyl-CoA-utilizing enzyme, then ACP recognition would have evolved at least three independent times (as shown by each clade in Fig. 2). An underlying assumption of molecular phylogeny is that the history with the least perceived changes is the true one [32]. Because of this, we employed motif analysis to ascertain how many times ACP-utilization evolved in this family.

### Analysis of ACP-utilization motifs

**Figure 4:**
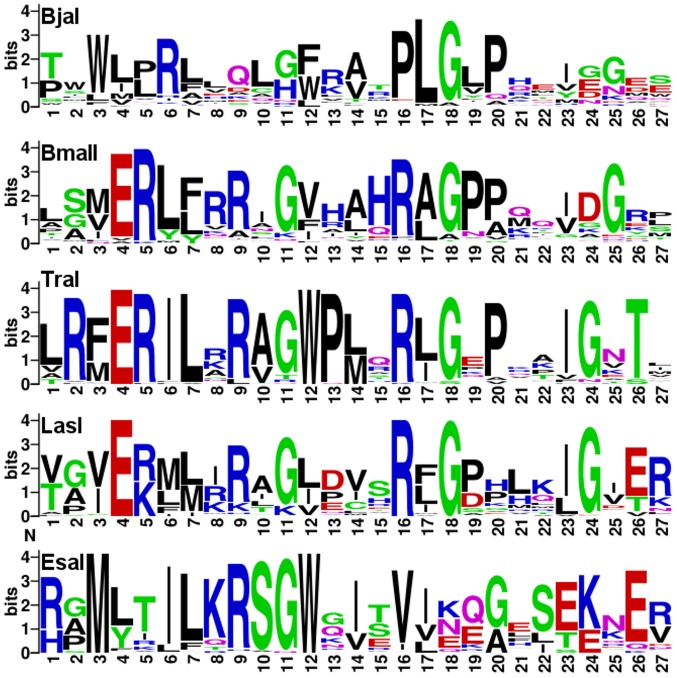
Protein logos of the ACP-binding loop for selected clades of acyl-HSL synthases. The clades are identified by a characterized member. The ACP binding region is based on a previously published analysis and corresponds to amino acid residues 146–173 of LasI and 144–172 of EsaI [19]. Positively charged residues are in blue.

We used motif analysis to examine regions involved in acyl-ACP-utilization. Structures of TofI, LasI and EsaI have a conserved surface helix and loop hypothesized to be involved in ACP recognition [19]. To examine the variations in this motif we took representatives of clades from the larger phylogeny (Fig. 2) and independently aligned sequences corresponding to the surface helix to obtain the resulting motifs (Fig. 4).

We can infer a number of things from the resulting motifs for each clade (Fig. 4). There is a notable absence of conserved positively charged residues in the aligned residues of the BjaI or acyl-CoA-utilizing clade. The only conserved positively charged residue is at position 6 and this position is not exposed in known structures. The motif analysis leads us to believe that BjaI should not interact with ACP strongly. The positively charged residue at position 9 is conserved in all clades with a characterized ACP-utilizing member, consistent with a significant decrease in activity with a mutation of this residue [19]. Compared to the LasI clade, the EsaI clade has some variations in positively charged residues, as was originally observed from the structures of the LasI and EsaI [19]. Because not all positively charged residues are conserved, it is unclear if EsaI-type ACP recognition evolved independently or diverged from the other groups in this analysis.

Overall, the BmaI1, TraI, and LasI clades have similar arrangements of positively charged residues (Fig. 4). This is consistent with ACP-utilization evolving once for the BmaI1-TraI-LasI clades and possibly a second time for the EsaI clade. The evolutionary history of the EsaI clade does not affect our conclusions regarding acyl-CoA-utilizing enzymes due to the fact that the EsaI clade is the least related to the BjaI clade (Fig. 2). Overall, our motif analysis supports our conclusion that CoA-utilizing acyl-HSL synthases evolved from ACP-utilizing ones.

### Kinetics of acyl-HSL synthases

We sought to investigate the acyl substrate specificity of these enzymes and how that relates the evolution of this protein family. To do this we determined kinetic parameters of two model enzymes: the isovaleryl-HSL synthase BjaI and the octanoyl-HSL synthase BmaI1 (Table 1). We confirmed and quantified that BjaI prefers isovaleryl-CoA as a substrate [15] whereas BmaI1 prefers octanoyl-ACP as a substrate [9]. We determined the kinetic constants for isovaleryl-CoA and isovaleryl-ACP with BjaI using a pseudo first-order analysis. We also determined the kinetic constants for octanoyl-ACP and octanoyl-CoA with BmaI1. These constants are combined with those for the *Pseudomonas aeruginosa* butyryl-HSL synthase, RhlI. The values for butyryl-ACP and butyryl-CoA with RhlI were found in another study using the same assay [33]. The Michaelis constants for isovaleryl-CoA and SAM are similar to Michaelis constants for SAM and butyryl-ACP for RhlI, although BjaI is an order of magnitude slower than RhlI [33], [34]. We note that the BjaI turnover rate is faster than the rates reported for *Agrobacterium tumefaciens* TraI or *Vibrio fischeri* LuxI [13], [14]. Regardless, it appears that the LuxI family acyl-HSL synthases are quite slow and acyl-HSL synthases are not under selection for catalytic efficiency.

**Table 1:** Kinetic constants for members of the acyl-HSL synthase family.

Enzyme	Substrate	*K*_m_ (µM)	*k*_cat_ (s^−1^)	*k*_cat_/*K*_m_ (s^−1^ M^−1^)	*k*_cat_/*K*_m_ ratio^b^
BjaI	Isovaleryl-CoA	7.0±0.5	0.021±0.03	3.0×10^3^	4.8×10^1^
BjaI	Isovaleryl-ACP	317±137	0.020±0.005	6.3×10^1^	
BmaI1	Octanoyl-CoA	541±14	0.0018±0.0002	3.3×10^0^	
BmaI1	Octanoyl-ACP	7.9±2	0.050±0.0008	6.3×10^3^	1.9×10^3^
RhlI^a^	Butyryl-CoA	200±22	0.050±0.002	1.4×10^2^	
RhlI	Butyryl-ACP	7.4±1.2	0.35±0.002	4.5×10^4^	3.2×10^2^

a RhlI kinetic constants are from another study [33].

b *k*_cat_/*K*_m_ ratio = (*k*_cat_/*K*_m_)^preferred substrate^/(*k*_cat_/*K*_m_)^non-preferred substrate^.

The ratio of *k*_cat_/*K*_m_ is a general measure of substrate activity with an enzyme. While comparing different substrates, the substrate with the higher *k*_cat_/*K*_m_ is the preferred one for an enzyme. From this we find that BjaI prefers acyl-CoAs whereas BmaI1 and RhlI prefer acyl-ACPs (Table 1). It appears all enzymes assayed have some ability to use both substrates. This would provide a means for the common ancestor to switch from acyl-ACP to acyl-CoA substrate utilization. We can look at the *k*_cat_/*K*_m_ of the preferred substrate divided by a nonpreferred substrate to quantifiy enzyme specificity. We find that BjaI discriminates the least between ACP and CoA substrates. This is consistent with an evolutionary history of substrate switching followed by use of an acyl group not known to be carried by an ACP.

### Using inhibitors to probe acyl-HSL substrate recognition

**Figure 5:**
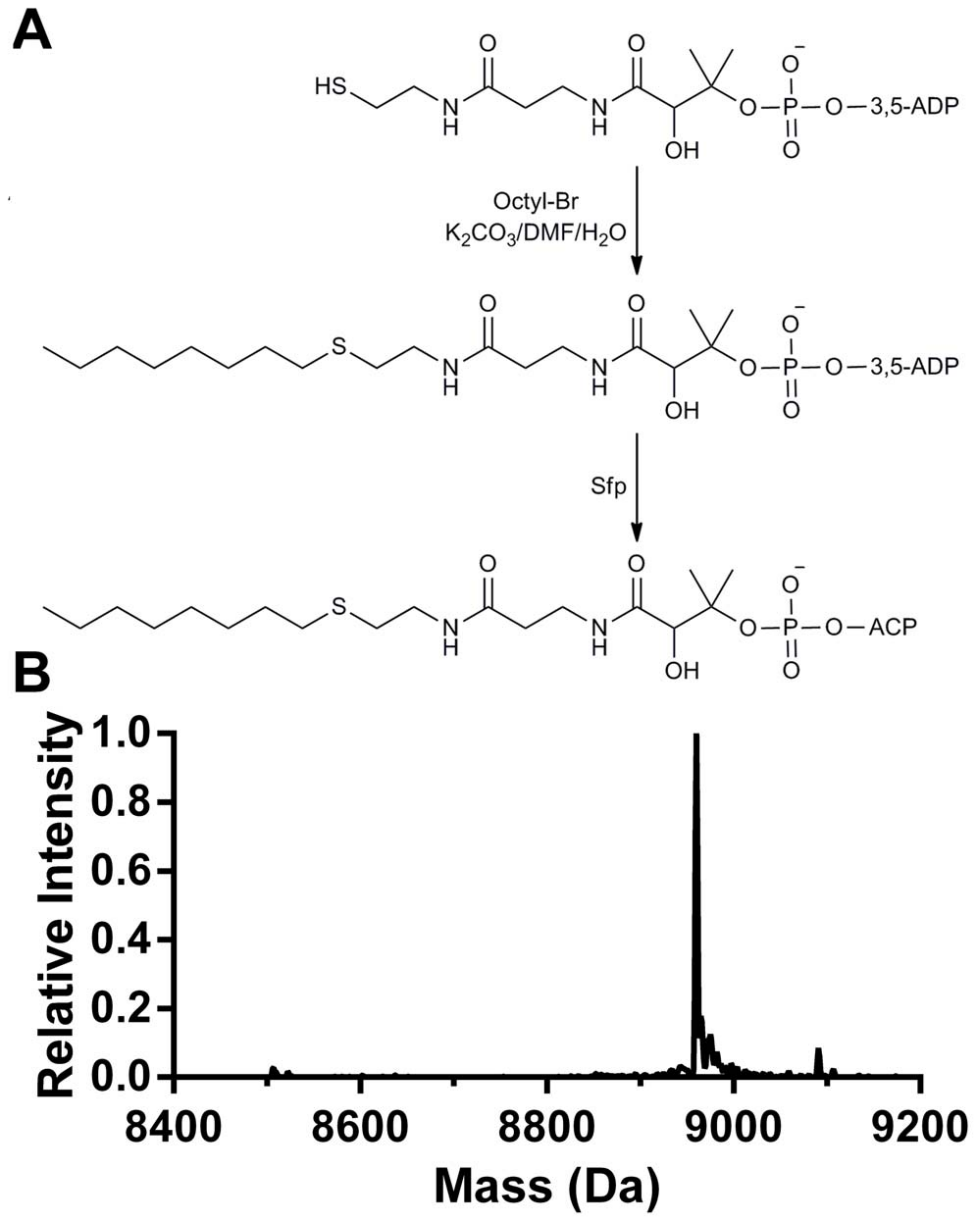
Chemoenzymatic synthesis of octyl-ACP sulfide. A) Synthesis of octyl ACP. In this two-step reaction, octyl-CoA sulfide was first synthesized by coupling octyl bromide with Coenzyme A, followed by enzymatic transfer of the alkyl-PPant to apo-ACP using *Bacillus subtilis* Sfp PPant transferase (see materials and methods). B) Mass spectrum of purified octyl-ACP. The intensity is relative to the largest peak of 8960 Da. The expected mass is 8957 Da.

**Figure 6:**
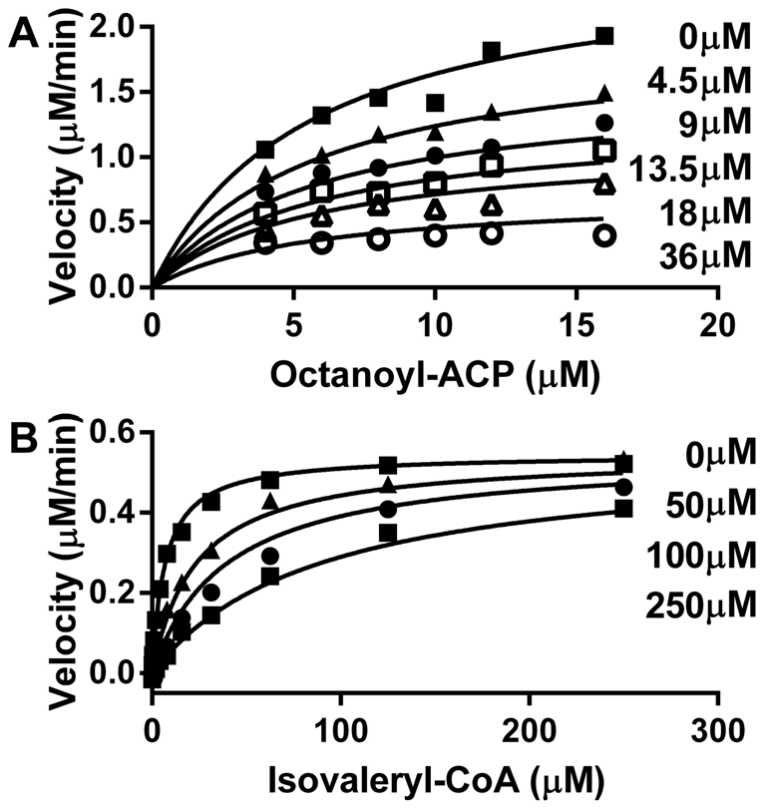
Inhibition of acyl-HSL synthases by substrate analogs. The best-fit models of inhibition are graphed. The µM concentration of inhibitor for each experiment is shown next to the curve. A) Substrate-velocity curves of mixed inhibition of 0.4 µM BmaI1 by octyl-ACP. B) Substrate-velocity curves of competitive inhibition of 0.5 µM BjaI with varying isopentyl-CoA.

To demonstrate recognition of the acyl-PPant moiety by acyl-HSL synthetases, we synthesized sulfide (thioether) analogs of the thioester substrates used by BjaI and BmaI1 and determined their inhibition constants (Table 2). We synthesized isopentyl-CoA thioether, an analog of isovaleryl-CoA (Fig. 5) and showed it competitively inhibits BjaI with respect to isovaleryl-CoA (Fig. 6). As the true substrate dissociation constant for isovaleryl-CoA is equal to or less than the Michaelis constant of 7 µM [35], this inhibitor binds to the enzyme less well because of the higher *K*_i_. We then synthesized the thioether analog of octanoyl-ACP, octyl-ACP (Fig. 5) and examined its ability to inhibit BmaI1 activity. We found octyl-ACP to be a noncompetitive (or mixed) inhibitor of BmaI1 with respect to octanoyl-ACP with an α (ratio of competitive to uncompetitive inhibition) of 0.3±0.2 (Fig. 6). The mixed inhibition of octyl-ACP indicates it binds to BmaI1 at more than one step of the reaction, or it binds to more than one enzyme form. We again find that the *K*_i_ is lower than the *K*_m_ for the analogous substrate. The higher inhibition constant relative to the substrate Michaelis constant is from reduced binding energy from the loss of the carbonyl oxygen and from the change of the carbonyl carbon from a sp2 to a sp3 configuration. The contribution of hydrogen bonding by the carbonyl oxygen would be consistent with the observed hydrogen bonding seen in the structure of *Burkholderia glumae* TofI bound to an acyl-substrate-like inhibitor [10]. The relative decrease in binding suggests the acyl-PPant moiety of the substrate is recognized by the enzyme. As the acyl-PPant moiety is shared by both acyl-CoA and acyl-ACP substrates (Fig. 1B), recognition of this could be the basis for substrate switching evolutionary events. This is consistent with the exaptation of acyl-PPant moiety for evolution of substrate recognition by this enzyme family.

**Table 2:** Kinetics of inhibition by sulfide analogs.

Enzyme	Inhibitor	Mode	Inhibitor *K*_i_ (µM)	Substrate *K*_m_ (µM)
BmaI1	Octyl-ACP	Noncompetitive	31±14	7.9±2.0
BjaI	Isopentyl-CoA	Competitive	21±1	7.0±0.5

## Discussion

LuxI-family acyl-HSL synthases are widely distributed among Proteobacteria, are useful components for synthetic biology, and are targets for novel antibacterial virulence therapies. We have recently learned that some LuxI family members utilize acyl-CoAs whereas others utilize acyl-ACPs as acyl donors [15]–[17]. The three known acyl-CoA-utilizing LuxI family members form a specific clade with several other uncharacterized LuxI family members (Fig. 2). We predict the uncharacterized members of this clade also prefer acyl-CoA substrates to acyl-ACP substrates. Both isovaleryl-CoA and isovaleryl-ACP share an acyl-PPant moiety, but BjaI prefers isovaleryl-CoA as a substrate (Table 1). The reduced activity of isovaleryl-ACP over isovaleryl-CoA with BjaI, which does not have an ACP-utilization motif, supports the hypothesis that this motif is important specifically for acyl-ACP use.

Our analysis of the natural evolution of this protein family is consistent with the view that acyl-CoA-utilizing LuxI homologs evolved from an ancestral acyl-ACP-utilizing acyl-HSL synthase (Fig. 2). The most parsimonious interpretation of the phylogeny is that acyl-CoA-utilizing acyl-HSL synthases evolved from an acyl-ACP-utilizing one. The similarity of the motifs from ACP-interacting regions also supports this conclusion (Fig. 4). We also find that the acyl-PPant moiety of these substrates is a common moiety and is important for substrate binding (Fig. 6, Tables 1 and 2), which is biochemically consistent with our evolutionary model. We consider the evolution of acyl-CoA-utilization from an acyl-ACP-dependent ancestor to represent a molecular exaptation as opposed to an adaptation. This is because the ancestor evolved to use ACP substrates but at some point utilized CoA substrates that were not selected for. Our combined phylogenetic and kinetic analyses provide evidence for an exaptation of acyl-PPant utilization from acyl-ACP to acyl-CoA utilization resulting in a new type of acyl-HSL synthase.

We can consider a model for this exaptation event in the light of what is known from other studies. Previous studies showed that, at high concentrations, butyryl-CoA serves as a poor substrate for the butyryl-ACP-dependent *Pseudomonas aeruginosa* RhlI [33], [34], and octanoyl-CoA can also serve as a poor substrate for BmaI1 (Table 1). On the other hand, we found that isovaleryl-ACP is a poorer substrate than isovaleryl-CoA for BjaI. These results agree with a model where the common ancestor of the clades containing BmaI1, RhlI, and BjaI possessed relaxed substrate specificity that eventually led to evolution of acyl-CoA-specificity. This is consistent with accepted models for evolution of new enzymes [21]–[23].

We consider exaptation of substrate recognition to be a general means for enzymes to evolve to use different acyl-PPant-containing substrates that could apply to other examples of substrate switching with shared moieties. In established enzyme evolutionary models, relaxed substrate specificity is a pre-existing property of an ancestral enzyme or arises through a period of neutral evolution in the absence of selection [21], [23]. In our study we find that analogous chemical moieties are a mechanism for preexisting relaxed substrate specificity. This renders a period of neutral evolution unnecessary in this case. Exaptation of substrate moiety recognition in enzyme evolution is a general mechanism for evolution of new enzymes.

## Materials and Methods

### Acyl-HSL synthase phylogeny

Protein sequences were aligned by using MUSCLE [36] and the edges of the alignment were trimmed with JalView [37] to remove regions with low conservation. Evolutionary analyses were conducted in MEGA5 [38]. The evolutionary history was inferred by using the Neighbor-Joining method [39]. The topology was similar when we used members of PF07395 or PF12746 as outgroups. The optimal tree with the sum of branch length 10.6 is shown. The percentages of replicate trees in which the associated taxa clustered together in the bootstrap test (1000 replicates) are shown next to the branches [40]. The tree is drawn to scale, with branch lengths in the same units as those of the evolutionary distances used to infer the phylogenetic tree. The evolutionary distances were computed using the p-distance method [41] and are in the units of the number of amino acid differences per site. The analysis involved 38 amino acid sequences. All ambiguous positions were removed for each sequence pair. There were a total of 259 positions in the final dataset.

### Logo construction

All protein sequences from PF00765 were obtained from Pfam. Sequences less than 160 amino acids and sequences with greater than 99% identity were removed with USEARCH [42]. The Mig14 family (PF07395) was added and sequences were aligned with MUSCLE [36]. Alignment edges were trimmed to give a uniform length as described above. A phylogeny was constructed from the alignment by using Fasttree [43]. Dendroscope [44] was used to visualize the phylogeny and select sequence labels for retrieval from Uniprot. Retrieved sequences were aligned with each other and LasI [36] and the ACP binding motif was selected with Jalview [37]. The LasI sequence was removed and a motif logo was constructed with Weblogo [45].

### Synthesis of alkyl-CoAs

Alkyl-CoA analogs were synthesized from alkyl-bromide and CoA using a modification of a previously published procedure [46]. 100 mg (0.13 mmol) of CoA was dissolved in a minimal mixture of 1∶1 dimethylformamide:water. To this mixture, 100 mg (0.52 mmol) 1-Bromoctane or 79.0 mg (0.52 mmol) 1-bromoisopentane was added along with 36.0 mg (0.26 mmol) of K_2_CO_3_. After gentle mixing, 32.5 mg (0.13 mmol) of TCEP was added to reduce any disulfide bonds. The reaction mixture was incubated overnight at room temperature with gentle stirring under a nitrogen environment. The mixture was then washed in a separatory funnel using diethyl ether to remove any organic contaminants. The aqueous layer was run through a Hypersep C18 column and filtered through a 44-µm filter. Alkyl-CoA was further purified by C18-reverse-phase HPLC with a gradient beginning at 98% buffer A (25 mM ammonium acetate at pH-5) and ending at 98% buffer B (acetonitrile) over a period of 25 min. The flow rate was 2 ml/min.

### Purification of proteins

*Burkholderia mallei* ATCC23344 BmaI1 was expressed from plasmid pQC201 [9] and *Bradyrhizobium japonicum* BjaI was expressed from pAL26 [15]. Both enzymes were purified by Ni-affinity chromatography as described for BmaI1 [9]. *Escherichia coli apo-*AcpP was purified by ion exchange and precipitation as described [47]. The 4′-PPant transferase from *Bacillus subtilis* 168 Sfp was expressed from plasmid pNRD136 [48] and was purified by Ni-affinity chromatography and precipitation as described [49]. Acyl-ACPs were synthesized using Sfp as described [49] using a 20∶1 ratio of acyl-CoA to ACP. ACPs were purified by precipitation and desalting using a GE Healthcare Lifesciences PD10 column.

### BjaI and BmaI1 activity assays

We measured BjaI activity by using a DCPIP microplate assay, with a 50 µL reaction volume in 384-well clear plates (Greiner 781185) similar to that described previously [9]. Reaction mixtures contained 50 mM HEPES (pH 7.5), 0.005% Nonidet NP40, 3.5 mM MES (pH 6.0), 7% glycerol, 100 µM DCPIP, 500 µM SAM-*p*-toluenesulfonate salt (Sigma A2408), 1 µM BjaI. The final pH of the reaction mixture was 7.3 and *p*-toluenesulfonate did not significantly affect BjaI reaction kinetics. For determination of kinetic constants, we varied the concentration of isovaleryl-CoA from 0 to 250 µM and isovaleryl-ACP from 0 to 500 µM. We found BjaI has an apparent Michaelis constant (*K*_m_) for SAM of 39±4 µM by varying it from 0 to 1 mM with 250 µM isovaleryl-CoA as a substrate. In inhibitor experiments, the concentration of isovaleryl-CoA substrate were varied from 0 to 250 µM and isopentyl-CoA inhibitor was varied from 0 to 250 µM. SAM and BjaI concentrations were maintained at 500 µM and 0.5 µM, respectively.

BmaI1 activity was also measured by using the DCPIP assay as described previously [9] in a buffer consisting of 100 mM HEPES, pH 7.2. While SAM concentration was kept at 3 M, BmaI1 was maintained at 0.5 µM and 5 µM during determination of kinetic constants for octanoyl-ACP and octanoyl-CoA substrates respectively. For the inhibitor assay, the concentrations of BmaI1 and SAM were maintained at 400 nM and 3 mM respectively. The concentration of octanoyl-ACP substrate was varied from 3 to 20 µM and octyl-ACP inhibitor varied from 0 to 36 µM. The final volume in each reaction mixture was 100 µl.

### Kinetic analyses

The apparent kinetic constants for substrates were obtained with Prism (Gaphpad software) by fitting the rate curve data to the Michaelis-Menten equation (equation 1).
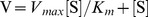
(1)


To determine apparent inhibition constants (*K_i_*), we fit substrate-velocity curves with different amounts of inhibitor to equations 2–5 described below [50]. The following equations that best fit according to the Akaike Information Criterion were reported.

Competitive inhibition:
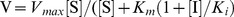
(2)


Noncompetitive inhibition:
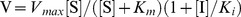
(3)


Uncompetitive inhibition:
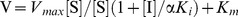
(4)


Mixed inhibition:
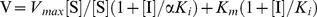
(5)


For fitting inhibition data, the *K_m_* for octanoyl-ACP with BmaI1 was set at 7.9 µM and the *K_m_* for isovaleryl-CoA with BjaI was set to 7 µM. We report the standard deviation from nonlinear regression replicates.
